# Triboelectric Bending Sensors for AI‐Enabled Sign Language Recognition

**DOI:** 10.1002/advs.202408384

**Published:** 2025-01-07

**Authors:** Wei Wang, Xiangkun Bo, Weilu Li, Abdelrahman B. M. Eldaly, Lingyun Wang, Wen Jung Li, Leanne Lai Hang Chan, Walid A. Daoud

**Affiliations:** ^1^ Department of Mechanical Engineering City University of Hong Kong Hong Kong China; ^2^ Department of Electrical Engineering City University of Hong Kong Hong Kong China; ^3^ School of Microelectronics Shandong University Jinan 250101 China; ^4^ Shenzhen Research Institute City University of Hong Kong Shenzhen 518000 China

**Keywords:** long short‐term memory, pattern recognition, smart wearable system, triboelectric bending sensor

## Abstract

Human–machine interfaces and wearable electronics, as fundamentals to achieve human‐machine interactions, are becoming increasingly essential in the era of the Internet of Things. However, contemporary wearable sensors based on resistive and capacitive mechanisms demand an external power, impeding them from extensive and diverse deployment. Herein, a smart wearable system is developed encompassing five arch‐structured self‐powered triboelectric sensors, a five‐channel data acquisition unit to collect finger bending signals, and an artificial intelligence (AI) methodology, specifically a long short‐term memory (LSTM) network, to recognize signal patterns. A slider‐crank mechanism that precisely controls the bending angle is designed to quantitively assess the sensor's performance. Thirty signal patterns of sign language of each letter are collected and analyzed after the environment noise and cross‐talks among different channels are reduced and removed, respectively, by leveraging low pass filters. Two LSTM models are trained using different training sets, and four indexes are introduced to evaluate their performance, achieving a recognition accuracy of 96.15%. This work demonstrates a novel integration of triboelectric sensors with AI for sign language recognition, paving a new application avenue of triboelectric sensors in wearable electronics.

## Graphical abstract

Herein, a smart wearable system is developed encompassing five self‐powered bending sensors, a five‐channel data acquisition unit to collect finger bending signals, and an artificial intelligence (AI) algorithm, specifically a long short‐term memory (LSTM) network, to recognize the signal patterns in real time. The designed system can achieve sign language recognition with an accuracy of 96.15%.

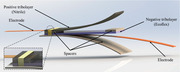

## 1 Introduction

The rapid advancement of micro‐nanotechnology and artificial intelligence (AI) has propelled tremendous progress of wearable electronics in the field of human motion tracking,^[^ ^1^, ^2^, ^3^, ^4^, ^5^, ^6^ ^]^ healthcare monitoring,^[^ ^7^, ^8^, ^9^, ^10^, ^11^, ^12^, ^13^ ^]^ and human–machine interfaces (HMI) and interactions.^[^ ^14^, ^15^, ^16^, ^17^, ^18^, ^19^, ^20^, ^21^, ^22^ ^]^ Among numerous wearable electronics, piezoelectric sensors, which generate a voltage at the opposite ends of a material surface when subjected to an external force, show great potential for use in wearable electronics.^[^ ^23^, ^24^, ^25^ ^]^ For example, Yuan et al. proposed a flexible bionic piezoelectric sensor for respiratory monitoring,^[^ ^26^ ^]^ and Li et al. designed a highly sensitive piezoelectric sensor for recording human joint movement and breathing.^[^ ^27^ ^]^ However, this kind of sensors output a small voltage and have a limited static voltage measurement capability.^[^ ^28^ ^]^ Therefore, triboelectric nanogenerators,^[^ ^29^, ^30^, ^31^ ^]^ which convert low frequency mechanical motions to a high voltage output without the aid of an external power, have been redeemed as a new generation of human‐oriented sensors that can be applied in wearable electronics.^[^ ^32^, ^33^, ^34^, ^35^ ^]^ The merits of self‐power, flexibility, lightweight, portability, low cost, effortless fabrication, and wide material choice have led to a growing interest in triboelectric based sensors. Wang et al. reported a highly sensitive self‐powered triboelectric angle sensor for recording the flexion/extension of joints.^[^ ^36^ ^]^ Anaya et al. proposed an eye motion detection system based on triboelectric interaction and near‐field transmission, which allows wireless near field remote sensing.^[^ ^37^ ^]^ Meng et al. developed a wireless textile‐based sensor system based on the triboelectric mechanism for personalized health care.^[^ ^38^ ^]^ Although these sensors can timely acquire and analyze the motion signals, they are unversed in grasping subtle signal information or mapping the relationship between human motion and the detected signals.

AI, versed in exploiting and extracting information from massive data, is able to distill comprehensive and fine‐grained information from the sensor's output. Therefore, many efforts have been devoted to develop smart HMI by combining machine learning algorithms with wearable electronics.^[^ ^39^, ^40^, ^41^, ^42^, ^43^, ^44^, ^45^ ^]^ Wen et al. designed a triboelectric smart glove to recognize sign language and communicate in VR space with the help of convolution neutral network, achieving an accuracy of 91.3% for words and 95% for sentences.^[^ ^46^ ^]^ Lu et al. reported a mouth triboelectric sensor to decode lip language by a dilated recurrent neural network with a 94.5% accuracy.^[^ ^47^ ^]^ Zhao et al. reported a speech recognition system using a triboelectric vibration sensor that can identify 17 commonly used expressions and recognize voice identity with an accuracy of 92.3% and 90.6%, respectively.^[^ ^48^ ^]^ Compared with the vision‐based recognition method,^[^ ^49^, ^50^, ^51^, ^52^, ^53^ ^]^ the action‐based recognition method is capable of recognizing dynamic hand gestures regardless of image partial occlusion and environmental illumination. However, the challenge of achieving a higher recognition accuracy in different scenarios still exists in practice despite the significant efforts made in improving the performance of triboelectric sensors and integrating them with machine learning algorithms.^[^ ^54^, ^55^, ^56^, ^57^, ^58^, ^59^, ^60^ ^]^

In this work, we develop a smart wearable system comprising five arch‐structured self‐powered triboelectric sensors, a microcontroller with embedded analog‐to‐digital converter (ADC), and an AI block to timely recognize the signal patterns of sign language of 26 letters. Five triboelectric bending sensors are first fabricated with nitrile film as the positive tribolayer and Ecoflex film as the negative tribolayer, where successive layer contacts and separations incurred by finger bending motions lead to voltage outputs based on the coupling of triboelectrification and electrostatic induction. A slider‐crank mechanism is then set up to accurately tune the bending angle and quantitatively evaluate the performance of the sensor. A sensitivity of ‐0.146 V deg^−1^ at 48.61° to 120° and −0.0128 V deg^−1^ at 20° to 48.61° is achieved along with response time of 490 ms (bending) and 420 ms (release), degree of hysteresis of 13.98%, and repeatability of 1000 bending cycles. Thirty signal patterns of sign language of each letter are collected through a customized five‐channel data acquisition unit, and a similarity analysis is carried out to quantitively assess the discrepancy of the 780 signal patterns. A long short‐term memory (LSTM) network with 256 hidden units is then trained by two different data groups, smoothing and original data. The model trained by the former achieves a recognition accuracy of 93.16%, while the latter achieves 96.15%. Finally, an application embedded the optimal LSTM model is developed to receive the sensor data and timely display the recognition result, where sign language recognition of letters A, B, C, and H are demonstrated. This AI‐assisted smart wearable system exhibits a promising approach to gesture monitoring, sign language translation, and human‐machine interaction.

## 2 Results

### 2.1 Triboelectric Bending Sensor Configuration and Working Mechanism

**Figure 1:**
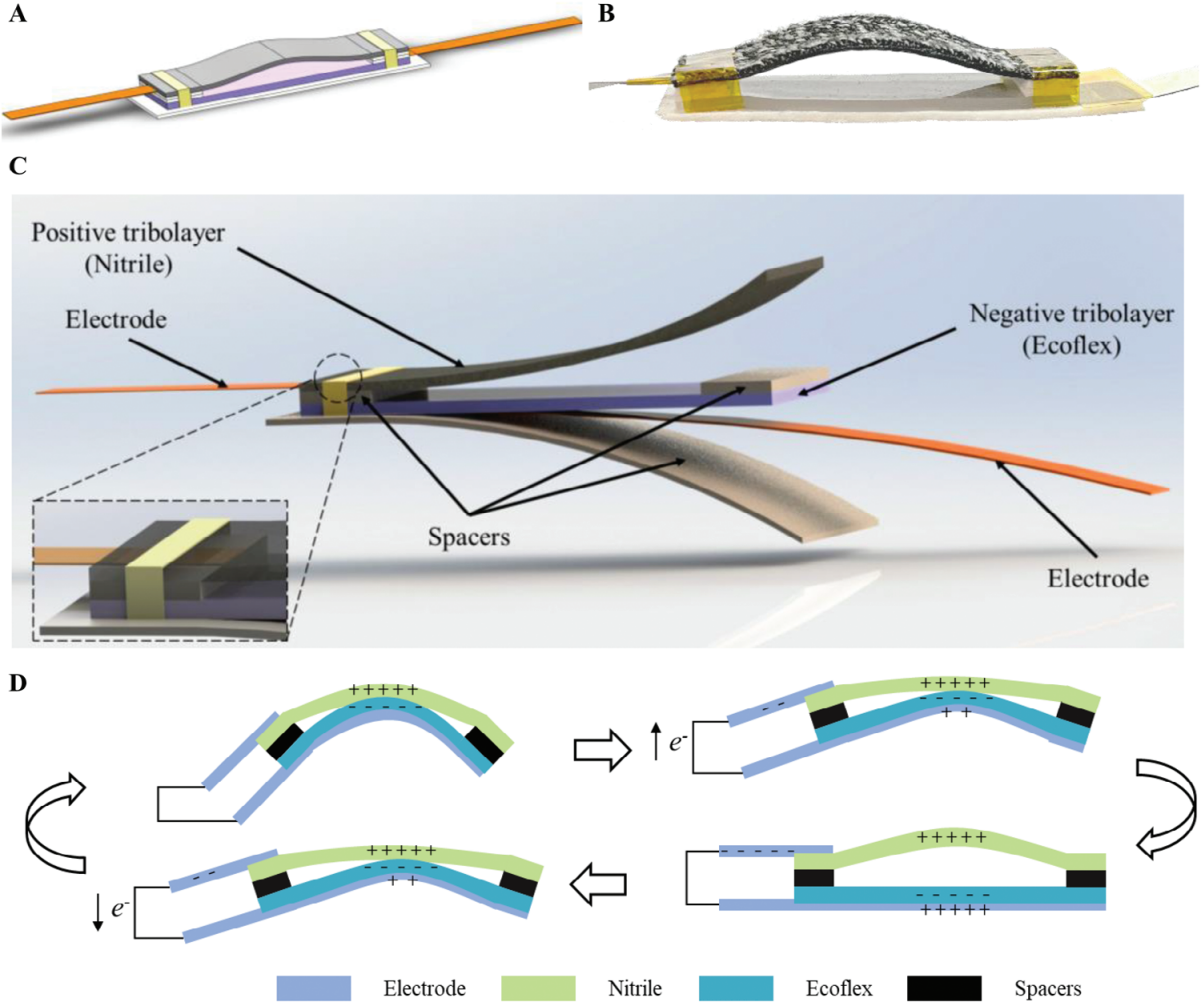
Triboelectric bending sensor configuration and working mechanism. a) Schematic illustration of the triboelectric sensor. b) A digital photograph of the fabricated sensor. c) Layer structure of the triboelectric bending sensor. d) Working mechanism of the sensor.

Triboelectric sensors can directly convert a low‐frequency motion into a voltage/current signal based on the effects of triboelectrification and electrostatic induction. In this study, five arch‐structured triboelectric sensors were fabricated for finger bending measurement (**Figure** **1**a,b). Details of the dimension and quantity of each component are provided in Table S1 (Supporting Information). The sensor installation on five fingers and the layout of the five triboelectric bending sensors are shown in Figure S1 (Supporting Information). A comparison of different device structures is provided in Table S2 (Supporting Information). The sensor mainly consists of a silicone rubber film as the negative tribolayer, a nitrile film as the positive tribolayer, and two pieces of conductive tape as the electrodes. Four pieces of nonconductive double‐sided foam tape and two piled pieces at the end are used to glue the positive tribolayer and negative tribolayer together. Two pieces of Kapton tape winded at the two ends are employed to further enhance the adhesion between the positive tribolayer, piled double‐sided foam tapes, and the negative tribolayer, preventing the components from disassembly in bending. A large rectangle double‐sided foam is used to fit the proximal interphalangeal joint or the interphalangeal joint of the thumb. The layer structure of the triboelectric bending sensor and surface profile of the positive tribolayer are shown in Figure 1c and Figure S2 (Supporting Information), respectively.

The working mechanism of the fabricated triboelectric sensors is articulated in Figure 1d. The initial state is defined as that the finger is horizontal and the two layers are fully separated. The first bending state, where the two layers are in full contact, is shown in the upper left‐side of Figure 1d, where equal positive and negative charges distribute on their surface due to the triboelectrification effect. No current or voltage difference is generated from the initial state to the first bending state as no directional movement of electrons occurs. The first release state follows the first bending state, and the two contacted layers gradually separate. In the separation process, positive charges are induced at the bottom electrode and negative charges are induced at the top electrode as a result of the electrostatic induction effect, generating a current and voltage difference. The separation process continues until the gap of the two layers reaches the maximum, i.e., the finger becomes horizontal. Next, the second bending cycle starts, and the positive tribolayer (nitrile) and negative tribolayer (Ecoflex) get closer. In this process, the electrons at the top electrode transfer to the bottom electrode to neutralize the remaining positive charges, generating an alternating current and a voltage difference. When the two layers fully contact together again, equal positive and negative charges distribute on the surface again, which is a repeat of the first bending state. Continuous finger bending and release lead to a periodic contact and separation of the two layers, generating voltage signals.

### 2.2 Characteristics of the Triboelectric Sensor

**Figure 2:**
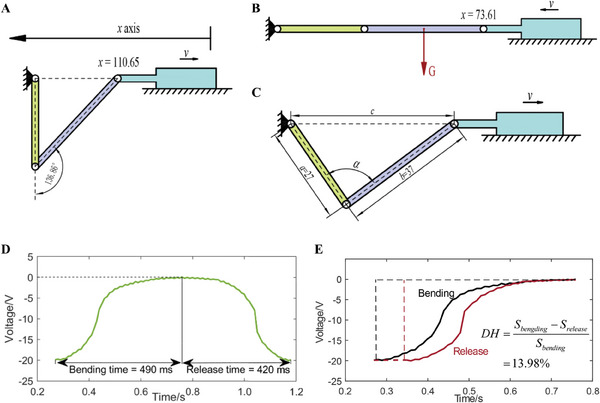
Response and hysteresis tests using the slider‐crank mechanism. a) Left limit and b) right limit position. c) Kinematic sketch of the slider‐crank mechanism. d) Response time of bending and release. e) Hysteresis of the triboelectric sensor.

A slider‐crank mechanism was designed and used to characterize the performance of the triboelectric sensor. The left limit position of the mechanism is shown in **Figure** **2**a, where the crank is vertical and the bending angle is 137°. The right limit position is shown in Figure 2b, where the three parts are in one line. As the driving force from the slider is perpendicular to the velocity of the crank, this mechanism cannot move unless the gravity *G* is considered. The forward and backward distance of the slider is precisely controlled by a linear motor, and thus the bending angle *β*, supplementary angle of *α*, is also precisely controlled as shown in Figure 2c. The quantitative relation between the moving distance *d* and the bending angle *β* is given as Equation (1):

(1) $$\begin{array}{c} d=a+b-c=a+b-\sqrt{a^{2}+b^{2}-2ab\cos\alpha }\\ =a+b-\sqrt{a^{2}+b^{2}+2ab\cos\beta } \end{array}$$

The tested bending angle, moving distance, and linear motor position are provided in Table S3 (Supporting Information), where the minimal bending angle at which the output can be detected is 20°. Figure S3 (Supporting Information) shows the experimental platform settings, and Video S1 (Supporting Information) shows the experiment of which the slider moves to the left limit position with an acceleration *a* = 0.5 m s^−2^.

**Figure 3:**
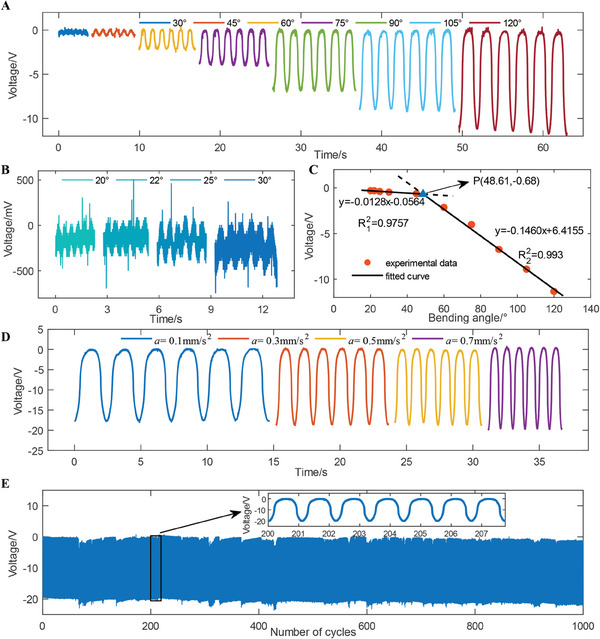
Characteristics of the triboelectric sensor. Voltage outputs dependence on a) large bending angle and b) small bending angle. c) Voltage response under different bending angles of experimental data and fitted curve. d) Voltage response at different accelerations. e) Cyclic testing of 1000 bending and release cycles.

The sensor performance, including the response time, hysteresis of one cycle, voltage output dependence on different bending angles and accelerations, and repeatability, was studied. As shown in Figure 2d, the response time of bending angle *β* = 137° is 490 ms for bending and 420 ms for release. The degree of hysteresis in one cycle, which is defined as a ratio of the area enclosed by bending and release curves (Figure 2e), is 13.98%. The material physical property causes the gap of bending and release time and therefore the hysteresis. The sensor outputs at different bending angles (20°, 22°, 25°, 30°, 45°, 60°, 75°, 90°, 105°, and 120°) are shown in **Figure** **3**a,b, where the voltage increases with bending angle increasing. Change of voltage response at a bending angle increment of 1° was tested. The results are shown in Figure S4 (Supporting Information). Large bending angles ranging from 45° to 120° lead to a significant voltage increase, and small bending angles ranging from 20° to 30° lead to a small increase in voltage.

A piecewise linear function with respect to the bending angel was determined through data fitting as shown in Figure 3c (standard deviation is shown in Table S4, Supporting Information). The slope represents the sensitivity, and thus the sensor exhibits a lower sensitivity (−0.0128 V deg^−1^) at small bending angle while a higher sensitivity (−0.146 V deg^−1^) at a large bending angle. The two fitted curves show determinate coefficients (*R* ^2^) of 0.976 and 0.993, respectively, and intersect at point P with a bending angle 48.61° and voltage ‐0.68 V. The voltage dependence on the forward and backward acceleration of the linear motor was tested to investigate the impact of bending frequency on the output. The results are shown in Figure 3d, where the voltage amplitudes of *a* = 0.3 and 0.5 are identical, indicating that the bending frequency has no significant impact on the output. Two slight increments are observed when the acceleration increases from 0.1 to 0.3 and from 0.5 to 0.7, for a higher bending frequency causes substantial charge accumulation on the surface of the two layers and the accumulated charges cannot be promptly dissipated. Cyclic testing was conducted to verify the repeatability. The result is shown in Figure 3e, which indicates the sensor output remains unchanged for over 1000 cycles of bending and release. Sensor's voltage response at different relative humidity (RH) was also investigated. The experiment setup is shown in Figure S5 (Supporting Information), where RH is controlled by a humidifier creating a moist environment (RH>60%) or N2 creating a dry environment (RH<60%), and RH of the small closed space is monitored by a hygrometer. As shown in Figure S6 (Supporting Information), the results indicate that the voltage response reaches a maximum at RH = 55%. A significant decrease from RH = 55% to RH = 65% and a small decrease from RH = 65% to RH = 75% were also observed. To quantitively describe the discrepancy in the sensing performance of the five sensors, we tested their voltage outputs at the bending angle of 75° using the slider‐crank mechanism. As shown in Figure S7 (Supporting Information), the average voltage response of sensors attached on thumb, index finger, middle finger, ring finger, and little finger is −3.62 V, −4.02 V, −4.11 V, −3.69 V, and −4.06 V, respectively. The maximal discrepancy in the sensing performance is 0.49 V.

### 2.3 Data Collection and Analysis

**Figure 4:**
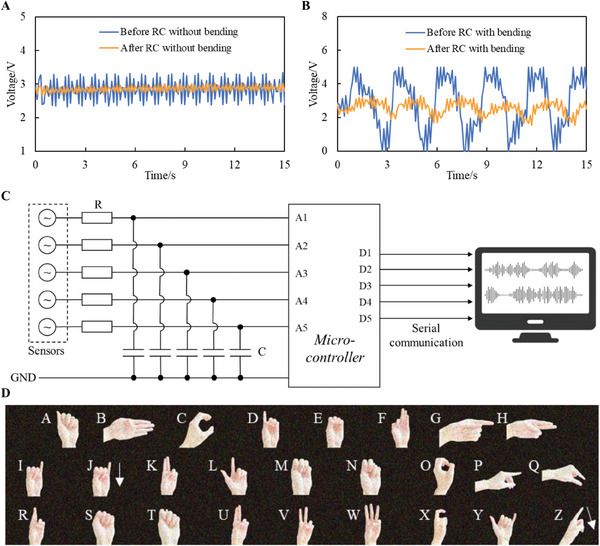
Data collection. a) Signals before and after the RC filter without bending. b) Signals before and after the RC filter with bending. c) Schematic of the five‐channel data acquisition unit. d) Sign language of 26 letters (left hand).

The environment noise should be filtered out at the time of collecting the finger bending signals, and therefore a low pass RC filter was designed and employed to increase the signal‐to‐noise ratio. The cut‐off frequency *ω*_c_ and transfer function *H*(*jω*) of a RC filter are determined by Equation (2):

(2) $$H\left(j\omega \right)=\frac{V_{o}}{V_{i}}=\frac{1}{1+j\omega /\omega_{c}}=\frac{1}{1+j\omega RC}$$

where *R* and *C* are resistance and capacitance, respectively. The schematic diagram of an RC filter and its output characteristic in frequency domain at three different cut‐off frequencies are shown in Figure S8 (Supporting Information). A much lower cut‐off frequency, *ω*_l_, leads to a narrow pass band and cannot filter out any noise. A far higher cut‐off frequency,* ω*_h_, causes a wide narrow pass band and filters out almost all the components including the desired signals, thus outputting a straight line without any information. Therefore, five 220 Ω resistors and five 280 pF capacitors are used to form five RC filters, and their performance without and with bending signals is respectively shown in **Figure** **4**a,b. In addition to the five bending sensors and RC filters, a five‐channel data acquisition unit, which can communicate with a PC through serial communication through the assistance of a microcontroller, is used. The system schematic is shown in Figure 4c, and the demonstration of collecting and visualizing data is shown in Video S2 (Supporting Information).

**Figure 5:**
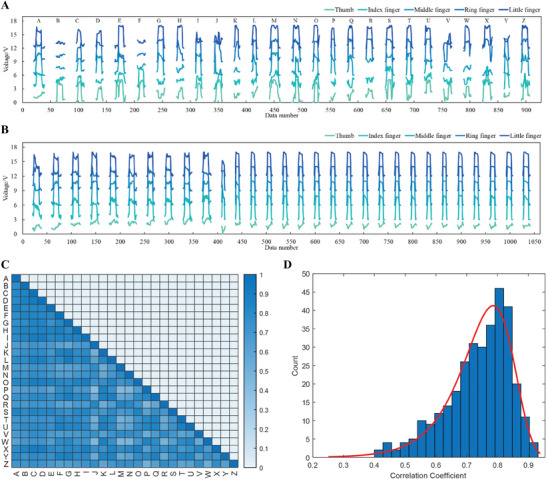
Data analysis. a) Signal patterns of the second sample of each letter. b) 30 signal patterns of letter A. c) CC matrix of 26 letters. d) Histogram of CC distribution with a fitted curve.

Twenty‐six hand gestures of letter sign language are shown in Figure 4d. Since a sign language of letter J and Z involves wrist rotations, which the sensor cannot sense, they are respectively redefined by the little and index finger bending. The end gesture of some letters is visually alike, e.g., M versus N and P versus Q, thus the bending sequences of these similar pairs (Table S5, Supporting Information) are defined for effortlessly discriminating their signal patterns. A database containing 30 signal patterns of each letter is attained for LSTM training and testing through the five‐channel data acquisition unit. **Figure** **5**a shows the signal patterns of the representative sample (No. 02) of each letter, where voltage shifts (0 V, 3 V, 6 V, 9 V, and 12 V) are respectively added to the output of thumb, index finger, middle finger, ring finger, and little finger to avoid curve overlap in each channel. Figure 5b shows 30 signal patterns of letter A. Data analysis (details in the methods section) is carried out to quantitively evaluate the similarity of different signal patterns. The analysis result of a lower triangular correlation coefficient (CC) matrix is shown in Figure 5c, and the value of each element is shown in Figure S9 (Supporting Information). The CC distribution with a fitted curve is derived (Figure 5d), which shows a negative skew distribution with the mean value of nondiagonal elements 0.7383. The data analysis indicates that these signal patterns exhibit a high similarity, indicating a significant obstacle of recognizing hand gestures in high accuracy. As a result, a more sophisticated tool is required to perfectly execute the pattern recognition task.

### 2.4 Pattern Recognitions Through LSTM

**Figure 6:**
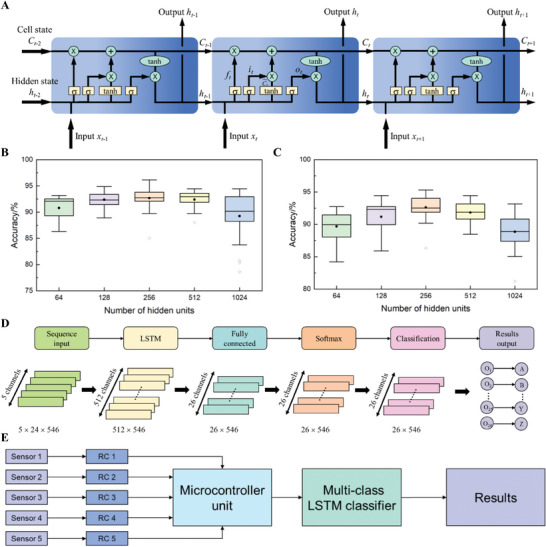
Pattern recognitions through LSTM. a) LSTM structure and mechanism. Box chart of accuracy variations under different hidden units with activation function of b) hard sigmoid and c) sigmoid. The box indicates 25–75% percentile; box middle lines indicate the median; filled circles in the box indicate the mean; whiskers indicate the range within 1.5 interquartile range (IQR); diamonds outside indicate the outliers. d) Configuration of the designed classifier. e) Diagram of the signal pattern recognition system.

LSTM is a recurrent neural network, which is widely used in time sequences processing, especially in speech recognition and optical character recognition (OCR) field.^[^ ^61^, ^62^ ^]^ The structure and mechanism of LSTM are shown in **Figure** **6**a, where forget gates, input gates, and output gates are introduced to control the passage of current state to the next unit. The mathematics of LSTM are articulated in the methods section. The impact of the number of hidden units and activation functions on LSTM performance was studied as shown in Figure 6b,c. The results indicate that the model performs the best at 256 hidden units with the activation function of hard sigmoid. Figure 6d shows the configuration of the designed classifier for pattern recognition, where the LSTM layer has 256 hidden units with state activation function tanh and gate activation function hard‐sigmoid. The input layer receives the data from the bending sensors, and then the LSTM layer recognizes the name of the input signal patterns. The following fully connected (FC) layer computes a weighted sum of LSTM outputs via Equation (3):

(3) $$Z=W^{T}X+B$$

where X and Z are the input and output of FC layer in a matrix form, respectively; W is the weighted matrix; B is the bias matrix. The next SoftMax layer maps the similarity, the outputs of FC layer to a probability through Equation (4), and finally the mapping results are outputted by a classification layer.

(4) $$p_{i}=\mathrm{softmax}\left(z_{i}\right)=e^{z_{i}}/\sum_{j=1}^{k}e^{z_{j}}$$

where *z_i_* is the *i*th output of the ahead FC layer; *p_i_* is the probability of *z_i_* belonging to a given category; *k* is the number of output nodes.

**Figure 7:**
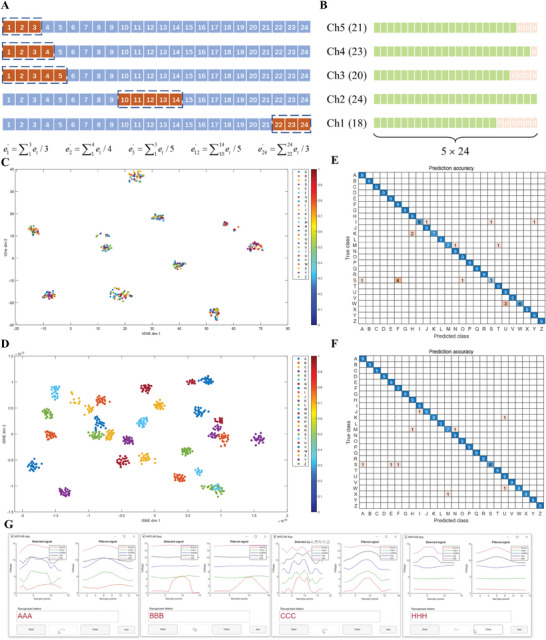
Pattern recognition results. a) Data smoothing through moving mean method. b) Zeros padding at the end of every sequence. Clustering results of c) input layer (data before LSTM) and d) output layer (data after LSTM). Confusion matrices of recognizing signal patterns using e) smoothing data and f) original data. g) Demonstration of recognizing letters A, B, C, and H.

Figure 6e shows the intact workflow of the signal pattern recognition system, where the trained LSTM classifier has 26 outputs of recognizing the sign language of 26 letters. Thirty signal patterns of each letter are divided into a training set covering 21 samples (70%) and a testing set covering nine samples (30%). Two different data groups are prepared to study the performance of the LSTM model. One group is the original data collected by the sensors, and the other group is the smoothing data where the original data is smoothed using a moving mean method whose window length is 5 as shown in **Figure** **7**a. Signal patterns of letter A before and after smoothing are shown in Figure S10. All the time series are then extended to a uniform length 24 by padding zeros at the end of every sequence (Figure 7b), before they are fed to the designed model. Seven key training arguments are provided in Table S6, and the training accuracy variations of using original data and smoothing data are shown in Figure S11 and S12, respectively.

To qualitatively evaluate the performance of the LSTM model, a clustering analysis is conducted using the t‐distributed stochastic neighbor embedding (t‐SNE) algorithm.^[^ ^63^, ^64^ ^]^ Figure 7c,d visualize the clustering results of the input layer (data before LSTM) and output layer (data after LSTM), respectively. The former shows considerable overlaps and a stochastic scatter among different categories, while the latter presents clear category boundaries with much less overlaps, indicating that the proposed LSTM model is seasoned and effective to recognize the signal patterns.

The recognition results of employing the two data groups to train the LSTM model are shown in Figure 7e,f, respectively. The two figures show that the smoothing data enables the model an accuracy of 93.16%, whereas the original data enables 96.15%, which outperforms most previous work as shown in Table S7 (Supporting Information). The other three indexes to evaluate model performance, precision, recall, and F‐score, are calculated as shown in Table S8 (Supporting Information). The LSTM model trained by smoothing data wrongly recognized 16 samples, including six samples of letter S, three samples of letter W, three samples of letter I, two samples of letter K, and two samples of letter M. Among the incorrect recognitions, four samples of S were classified to F, two samples to A and O, while three samples of W were classified to U. Three samples of I were misclassified to J, S, and Y, and two samples of M were misclassified to N and T. In contrast, the model trained by original data misrecognizes only 9 samples without duplicated misestimation. As shown in Figure 7f, three samples of letter S were misestimated to A, E, and F; two samples of letter M were misestimated to H and N; one sample of X, one sample of W, one sample of K, and one sample of J were misestimated to M, U, U, and I, respectively. Therefore, both of the two models perform well in recognizing sign language of letters, although they are not skilled enough to 100% correctly recognize signal patterns of letter M or S. The higher recognition accuracy performed by the second LSTM model indicates that the original data contains more learnable features than smoothing data and that the moving mean method partially eliminates some signal features, such as the tiny spikes and inflection points. The overall performance is compared in Figure S13 (Supporting Information). To demonstrate the functionality of the system, a MATLAB application was developed with the optimal trained LSTM model embedded. Successive hand gestures representing letters A, B, C, and H for three times were made. Meanwhile, the customized data acquisition unit received and transmitted the sensor outputs to the MATLAB application in real time. In the application, the collected data was fed into the embedded LSTM model, and the model recognized the input signal patterns and outputted a result (Figure 7g and Video S3, Supporting Information). This demonstration shows an interactive scenario from sensor operation and data collection to real‐time recognition.

## 3 Conclusion

In this work, five arch‐structured triboelectric bending sensors were fabricated, and their performance was studied using a slider‐crank mechanism. The results showed a fast response (490 ms for bending and 420 ms for release), lower hysteresis (13.98%), good sensitivity (−0.146 V deg^−1^ for large bending angle and −0.0128 V deg^−1^ for small bending angle), and high repeatability (over 1000 cycles). A five‐channel data acquisition unit was developed by integrating the sensor with RC filters and a microcontroller with embedded ADC. Signal patterns of sign language of 26 letters were collected. Data analysis was carried out and the results showed a high similarity among these signal patterns. Pattern recognition of sign language of 26 letters was achieved through LSTM network. The model trained by smoothing data reached a recognition accuracy of 93.16% and the other trained by original data reached 96.15%, which was higher than most literature works. An application of timely collecting and recognizing signal patterns was demonstrated. This work showed a creative integration way of self‐powered sensors and AI methodology in sign language recognition with a higher correct rate, paving a new application avenue of triboelectric sensors and widening the application scenarios of wearable electronics. Potential limitations of employing machine learning algorithm to recognize signal patterns include model generalizability and data drifting particularly when deploying the well‐trained model to recognize new inputs whose distribution is dissimilar from the training set. The two problems may incur that the model cannot recognize some signal patterns that are not included in the training set in a high accuracy. In the future, the generalizability of the designed model could be further improved by adding more data from different subjects and testing environment on the training set, and data drifting could be addressed by introducing a drift detection and a drift adaptation component.^[^ ^65^ ^]^

## 4 Experimental Section

### Triboelectric Sensor Fabrication

The negative tribolayer, silicone rubber film, was prepared by mixing 1 mL part A and 1 mL part B of Ecoflex 00–50 in a culture dish (diameter: 6 cm). Then, the culture dish was placed on an experimental table for one hour to solidify, forming silicone rubber film. The positive tribolayer, nitrile film, was directly cut and obtained from commercial nitrile gloves. To assemble the device, silicone rubber, nitrile film, conductive tape, and double‐sided foam tape were cut into different design size as shown Table S1 (Supporting Information). Then one piece of conductive tape was placed at the bottom of nitrile film as the left‐side electrode, and the two films were attached by two pieces of double‐sided foam tape, where the two ends of the silicone rubber film were aligned with that of nitrile, achieving a middle‐arched architecture of the nitrile film (Figure 1a,b). Next, the other piece of the conductive tape was placed at the bottom of the silicon rubber film as the right‐side electrode. Finally, two pieces of Kapton tape winded the two films together on the two ends, followed by attaching a rectangle double‐sided foam tape at the bottom of silicon rubber film. Five identical triboelectric sensors were fabricated and were attached on the five fingers.

### Sensor Output Characterization

The slider‐crank mechanism was fabricated by 3D printing (filament: PLA). The slider was driven by a linear motor (LinMot PS01‐23×160‐R). The voltage output of the triboelectric sensor was measured through an electrometer Keithley 6514 and a low‐noise current preamplifier (Model SR570).

### Data Collection and Dataset Configuration

The participant of the sign language gestures should be at the age of 20–30 and the experiment was conducted at a temperature of 23–26 °C and a relative humidity of 65%‐70%. The data was collected by a five‐channel data acquisition unit. Printed circuit board Arduino Uno Rev3 was employed as the microcontroller. Through repeating each finger gesture 30 times, 30 samples of each letter and a total of 780 samples were obtained. Seventy percent samples (21) were used for training and the rest 30% samples (9) were used for testing.

### Similarity Analysis of Triboelectric Signals

Pearson correlation coefficient (CC) was introduced to evaluate the similarity of different signal patterns. It was defined as the ratio of covariance to standard variance and calculated by Equation (5):

(5) $$\rho_{X,Y}=\frac{cov\left(X,Y\right)}{\sigma_{X}\sigma_{Y}}=\frac{E\left[\left(X-\mu_{X}\right)\left(Y-\mu_{Y}\right)\right]}{\sigma_{X}\sigma_{Y}}$$

where *μ* is the mean and *σ* is the standard variance; *X* and *Y* are two observed variables. When two variables variate identically, i.e., both increasing or decreasing in the same direction, the product (*X*‐*μ*_X_)*(*Y*‐*μ*_Y_) is always positive as the two terms have the same sign (both positive or negative), leading to a positive *ρ*_X,Y_. As a result, a positive value indicates that the two variables fluctuate identically, and a negative value indicates they fluctuates conversely. Further, a large absolute value indicates a strong correlation between the two variables. In this study, each signal pattern comprised five features from five channels, and thus the dimension of an individual CC matrix of two signal patterns was 5 by 5, where the element *ij* represented the *ρ*_X,Y_ between the *i*th feature of the first signal pattern and the *j*th feature of the second signal pattern.

(6) $$\rho_{X,Y}=\left[\begin{array}{c} \begin{array}{cccc} a_{11} & \;a_{12} & \;a_{13} & \;\begin{array}{cc} a_{14} & \;a_{15} \end{array}\\ a_{21} & \;a_{22} & \;a_{23} & \;\begin{array}{cc} a_{24} & \;a_{25} \end{array}\\ a_{31} & \;a_{32} & \;a_{33} & \;\begin{array}{cc} a_{34} & \;a_{35} \end{array}\\ a_{41} & \;a_{42} & \;a_{43} & \;\begin{array}{cc} a_{44} & \;a_{45} \end{array} \end{array}\\ \begin{array}{cccc} a_{51} & \;a_{52} & \;a_{53} & \;\begin{array}{cc} a_{54} & \;a_{55} \end{array} \end{array} \end{array}\right]$$

The diagonal elements (cases of *i* = *j*) of the individual CC matrix were the correlations of the same feature, and thus their average, *cc*, was employed to evaluate the similarity of the two signal patterns.

(7) $$cc\left(X,Y\right)=\frac{1}{5}\sum_{n=1}^{5}a_{nn}$$

Since each signal pattern had 30 samples, the *cc* mean of 30 samples was calculated as the nondiagonal elements of Figure 3g and Figure S3 (Supporting Information).

(8) $$CC\left(X,Y\right)=\frac{1}{30}\sum_{k=1}^{30}cc_{k}\left(X,Y\right)$$

*Mathematics of LSTM*: In Figure 4a, each LSTM unit has three inputs (*C*_t‐1_, *h*_t‐1_, *x*_t_) and two outputs (*C*_t_, *h*_t_) flowing to the next unit. The activation vector of the output gate *o*_t_ is defined as follows:

(9) $$o_{t}=\sigma \left(\left[W_{o},U_{o}\right]\left[\begin{array}{c} x_{t}\\ h_{t-1} \end{array}\right]+b_{o}\right)$$

where *σ* is the activation function, and *sigmoid* function is usually used; *W_o_* and *U_o_* are the weight matrix of the input and weight matrix of the recurrent connections for the output gate, respectively; *x*_t_ is the input at current time step; *h*_t‐1_ is the hidden state vector/output vector at time step t‐1; *b_o_* is the bias vector of the output gate. The hidden state vector/output vector *h*_t_ is defined as follows:

(10) $$h_{t}=o_{t}\times \tanh\left(C_{t}\right)$$

where *C*_t_ is the cell state vector at current time step, and it is defined as below:

(11) $$C_{t}=f_{t}\times C_{t-1}+i_{t}\times {\tilde{C}}_{t}$$

where *f*_t_ is the activation vector of the forget gate, and it is defined as below:

(12) $$f_{t}=\sigma \left(\left[W_{f},U_{f}\right]\left[\begin{array}{c} x_{t}\\ h_{t-1} \end{array}\right]+b_{f}\right)$$

where *W*_f_ and *U*_f_ are the weight matrix of the input and weight matrix of the recurrent connections for the forget gate, respectively; *b*_f_ is the bias vector of the forget gate. *i*_t_ in Equation (11) is the activation vector of the input gate and controls which feature is used to update the cell state. It is given as follows:

(13) $$i_{t}=\sigma \left(\left[W_{i},U_{i}\right]\left[\begin{array}{c} x_{t}\\ h_{t-1} \end{array}\right]+b_{i}\right)$$

where *W*_i_ and *U*_i_ are the weight matrix of the input and weight matrix of the recurrent connections for the input gate, respectively; *b*_i_ is the bias vector of the input gate. ${\tilde{C}}_{t}$ in Equation (11) is the cell input activation vector, and it is defined as below:

(14) $${\tilde{C}}_{t}=\tanh\left(\left[W_{C},U_{C}\right]\left[\begin{array}{c} x_{t}\\ h_{t-1} \end{array}\right]+b_{C}\right)$$

where *W*_C_ and *U*_C_ are the weight matrix of the input and weight matrix of the recurrent connections for the cell input activation vector, respectively; *b*_C_ is the bias vector of the cell input activation vector. The unknown eight weight matrices and four bias vectors were learned by training the LSTM network in MATLAB R2022b.

### Letter Recognition Demonstration

The five‐channel signal acquisition unit collected the raw data of different finger gestures and transmitted it to the developed MATLAB application via serial communication. In the application, the deployed well‐trained LSTM network received the signals and displayed the recognition result.

### Statistical Analysis

All data are representative of the samples. Data pre‐processing procedure of establishing the smoothing dataset is elaborated in Figure 7a,b. Standard deviation of the experimental data points in Figure 3c is provided in Table S4 (Supporting Information). Details of interval confidence calculation are provided at the end of Table S8 (Supporting Information). The sample size for LSTM training and testing is provided in the subsection “Data Collection and Dataset Configuration”. The software used for statistical analysis are MATLAB, Origin Lab, and Excel.

## Conflict of Interest

The authors declare no conflict of interest.

## Author Contributions

W.A.D., L.W., and W.W. conceived the initial concept. W.W. planned and conducted the experiment, data analysis, LSTM recognition, and drafted the manuscript. X.B. designed the sensor structure. W.L. and W.J.L. assisted in the sensor performance testing and evaluation. L.L.H.C. assisted in the LSTM performance evaluation. A.B.M.E. assisted in the machine learning analysis. W.A.D. supervised the project and revised the manuscript.

## Supporting information



Supporting Information

Supplemental Video 1

Supplemental Video 2

Supplemental Video 3

## Data Availability

The data that support the findings of this study are available from the corresponding author upon reasonable request.s
