# Supplementary material for: Triboelectric Bending Sensors for AI‐Enabled Sign Language Recognition
Source: Adv Sci (Weinh). 2025 Jan 7;12(8):2408384. doi: 10.1002/advs.202408384 (PMC11848593; doi:10.1002/advs.202408384)
Supplement: Supplementary file 1 — Supporting Information [file ADVS-12-2408384-s004.docx]

Supporting Information

**Triboelectric Bending Sensors for AI Enabled Sign Language Recognition**

Wei Wang^1^, Xiangkun Bo^1^, Weilu Li^1^, Abdelrahman B.M. Eldaly^2^, Lingyun Wang^3^, Wen Jung Li^1^, Leanne Lai Hang Chan^2^, Walid A. Daoud^1,4,*^

^1^Department of Mechanical Engineering, City University of Hong Kong, Hong Kong, China

^2^Department of Electrical Engineering, City University of Hong Kong, Hong Kong, China

^3^School of Microelectronics, Shandong University, Jinan 250101, China

^4^Shenzhen Research Institute, City University of Hong Kong, Shenzhen 518000, China

*Correspondence: wdaoud@cityu.edu.hk

**This file includes:**

Table S1 to S8

Figure S1 to S13

References [1-32]

Table S1. Dimension of each part of fabricated sensor.

| **Parts** | **Dimension/cm** | **Quantity** |
| --- | --- | --- |
| Silicon rubber | 4×1.5×0.17 | 1 |
| Nitrile | 4.3×1.5×0.13 | 1 |
| Conductive tape | 7.5 (L), 11 (R) | 2 |
| Mediate double-sided foam tape | 1.5×0.5×0.1 | 4 |
| Bottom double-sided foam tape | 4.5×2×0.1 | 1 |

Table S2. Comparison of different device structures.

| **Structure** | **Reference** | **Advantage** |
| --- | --- | --- |
| Plane structure | [1][2][3][4] | Compact and reliable |
| Arched structure | [5][6][7][8][9][10] | Full contact and separation |
| Yarn-like structure | [11][12][13] | High sensitivity |
| Wavy-like structure | [14][15] | Work at compressive and stretching mode |

The curved design maximizes the electrification area, thus enhancing the efficiency of charge transfer. During finger bending and release, the arched layer helps achieve a complete contact and separation cycle^[16,17]^. In addition, the arched structure shows low fatigue and failure likelihood over time as the curved design helps distribute mechanical stress more evenly and therefore the device can better bear bending forces, making the device more durable.

Table S3. Details of tested bending angle and linear motor position.

| **Bending angle *β* /°** | **Moving distance *d* /mm** | **Linear motor position (*x*-axis)** |
| --- | --- | --- |
| 120 | 30.85 | *x* = 104.46 |
| 105 | 24.24 | *x* = 97.85 |
| 90 | 18.2 | *x* = 91.81 |
| 75 | 12.86 | *x* = 86.47 |
| 60 | 8.35 | *x* = 81.96 |
| 45 | 4.75 | *x* = 78.36 |
| 30 | 2.13 | *x* = 75.74 |
| 25 | 1.48 | *x* = 75.09 |
| 22 | 1.15 | *x* = 74.76 |
| 20 | 0.95 | *x* = 74.56 |
| 15 | 0.53 | *x* = 74.14 (No signal received) |

Table S4. Standard deviation of experimental data points in Figure 3c.

| Angle/deg | 20 | 22 | 25 | 30 | 45 | 60 | 75 | 90 | 105 | 120 |
| --- | --- | --- | --- | --- | --- | --- | --- | --- | --- | --- |
| STD | 0.0260 | 0.0155 | 0.0161 | 0.0163 | 0.0148 | 0.0163 | 0.0365 | 0.0710 | 0.0275 | 0.0750 |

Table S5. Letter pairs and bending sequence.

| **Pair No.** | **Letters** | **Bending sequences** |
| --- | --- | --- |
| i | I | 1) ring, middle, and index; 2) thumb |
|  | J | 1) ring, middle, and index; 2) thumb; 3) little |
| ii | M | 1) thumb and little; 2) ring, middle, and index |
|  | N | 1) thumb, ring, and little; 2) middle and index |
| iii | P | ~30 degree bending of ring, middle, and thumb |
|  | Q | ~90 degree bending of ring, middle, and thumb |
| iv | R | ~1 second holding time |
|  | U | 1) ring and little; 2) thumb |
|  | V | 1) ring; 2) thumb; 3) little |

Table S6. Key training arguments for training the classifier.

| Max epochs | Batch size | Slover | Learning rate | Sequence length | Gradient threshold |
| --- | --- | --- | --- | --- | --- |
| 400 | 182 | Adam | 0.01 | longest | 1 |

Table S7. Comparison with reported works in gesture recognition.

| **Literature** | **Gestures** | **Sensor** | **Algorithm** | **Accuracy** |
| --- | --- | --- | --- | --- |
| Ref [4] | 50 ASL words and 20 sentences | Triboelectric sensor | CNN | 91.3% (50 words) and 95% (17 sentences) |
| Ref [10] | 11 flower management gestures | Triboelectric sensor | CNN | 95.23% |
| Ref [18] | 10 Chinese number gestures, 5 finger gestures, and 6 wrist gestures | Barometric pressure sensor | LDA | 90.21%, 94.4%, and 98.05% |
| Ref [19] | 72 CSL hand gestures and 24 ASL letters | IMU | DFFN | 96.1% and 99.93% |
| Ref [20] | 28 gestures (26 ASL letters + 2 signs) | Resistive flex sensor, IMU, and pressure sensor | SVM | 98.2% |
| Ref [21] | 20 words of lip motion | Triboelectric sensor | Dilated RNN | 94.5% |
| Ref [22] | 11 ASL hand gestures (6 letters, 4 numbers, and 1 sentence | Triboelectric yarn sensor | Multi-class SVM | 98.63% |
| Ref [23] | 26 ASL letters | Resistive yarn sensor | ANN | 99.8% |
| Ref [24] | 40 signs (26 ASL alphabets + 14 words) | Resistive flex sensor and IMU | CNN | 82.19% (static gestures) and 97.35% (dynamic gestures) |
| Ref [25] | 27 words and 27 ASL alphabets (26 letters + one for  home position) | Myo armband (sEMG + IMU) | SVM | 79.35% |
| Ref [26] | 13 ASL words | Myo armband (sEMG + IMU) | ANN, SVM, and HMM | 93.79%, 85.9%, and 85.56% |
| Ref [27] | 26 ASL letters | Triboelectric sensor and piezoelectric sensor | LDA | 92.6% |
| Ref [28] | 48 CSL words and 10 sentences | Resistive strain sensor | CNN | 95.8% and 84% |
| Ref [29] | 72 CSL words and 40 sentences | Accelerometer and EMG | DT and multi-stream HMM | 95.8% (words) and 93.1% (sentences) |
| Ref [30] | 10 ASL numbers | Resistive pressure Sensors | ELM | 93% |
| Ref [31] | 5 types of spherical objects | E-skin (triboelectric nanogenerator) | LSTM | 97.2% |
| Ref [32] | 9 numbers (1-9) | Triboelectric sensor array | LDA | 95% |
| **This work** | **26 ASL letters** | **Triboelectric sensor** | **LSTM** | **96.15%** |

Table S8. Classification performance of the two models.

| **Smoothing data** | | | | **Original data** | | | |
| --- | --- | --- | --- | --- | --- | --- | --- |
| Gesture | Precision | Recall | F-score | Gesture | Precision | Recall | F-score |
| A | 90.00% | 100.00% | 94.74% | A | 90.00% | 100.00% | 94.74% |
| B | 100.00% | 100.00% | 100.00% | B | 100.00% | 100.00% | 100.00% |
| C | 100.00% | 100.00% | 100.00% | C | 100.00% | 100.00% | 100.00% |
| D | 100.00% | 100.00% | 100.00% | D | 100.00% | 100.00% | 100.00% |
| E | 100.00% | 100.00% | 100.00% | E | 90.00% | 100.00% | 94.74% |
| F | 69.23% | 100.00% | 81.82% | F | 90.00% | 100.00% | 94.74% |
| G | 100.00% | 100.00% | 100.00% | G | 100.00% | 100.00% | 100.00% |
| H | 81.82% | 100.00% | 90.00% | H | 90.00% | 100.00% | 94.74% |
| I | 100.00% | 66.67% | 80.00% | I | 90.00% | 100.00% | 94.74% |
| J | 90.00% | 100.00% | 94.74% | J | 100.00% | 88.89% | 94.12% |
| K | 100.00% | 77.78% | 87.50% | K | 100.00% | 88.89% | 94.12% |
| L | 100.00% | 100.00% | 100.00% | L | 100.00% | 100.00% | 100.00% |
| M | 100.00% | 77.78% | 87.50% | M | 87.50% | 77.78% | 82.35% |
| N | 90.00% | 100.00% | 94.74% | N | 90.00% | 100.00% | 94.74% |
| O | 90.00% | 100.00% | 94.74% | O | 100.00% | 100.00% | 100.00% |
| P | 100.00% | 100.00% | 100.00% | P | 100.00% | 100.00% | 100.00% |
| Q | 100.00% | 100.00% | 100.00% | Q | 100.00% | 100.00% | 100.00% |
| R | 100.00% | 100.00% | 100.00% | R | 100.00% | 100.00% | 100.00% |
| S | 75.00% | 33.33% | 46.15% | S | 100.00% | 66.67% | 80.00% |
| T | 90.00% | 100.00% | 94.74% | T | 100.00% | 100.00% | 100.00% |
| U | 75.00% | 100.00% | 85.71% | U | 81.82% | 100.00% | 90.00% |
| V | 100.00% | 100.00% | 100.00% | V | 100.00% | 100.00% | 100.00% |
| W | 100.00% | 66.67% | 80.00% | W | 100.00% | 88.89% | 94.12% |
| X | 100.00% | 100.00% | 100.00% | X | 100.00% | 88.89% | 94.12% |
| Y | 90.00% | 100.00% | 94.74% | Y | 100.00% | 100.00% | 100.00% |
| Z | 100.00% | 100.00% | 100.00% | Z | 100.00% | 100.00% | 100.00% |

| Mean | 93.89% | 93.16% | 92.58% | Mean | 96.51% | 96.15% | 96.05% |
| --- | --- | --- | --- | --- | --- | --- | --- |
| Lower CI | 90.32% | 86.99% | 88.11% | Lower CI | 94.38% | 92.97% | 94.00% |
| Upper CI | 97.46% | 99.33% | 97.05% | Upper CI | 98.65% | 99.34% | 98.10% |

$$\begin{matrix} Precision=\frac{TP}{TP+\sum FP} \\ Recall=\frac{TP}{TP+\sum FN} \\ F-score=\frac{2*P*R}{P+R} \end{matrix}$$

$$Confidence intervals\text{ (}CI)=\bar{x}\pm z_{\alpha/2}\frac{\sigma}{\sqrt{n}}$$

where *TP* is true positive that the positive sample is correctly identified; *FP* is false positive that the positive sample is wrongly identified; *FN* is false negative that the negative sample is incorrectly identified; *P* and *R* are precision and recall, respectively; $\bar{x}$ is the sample mean; *z*_α/2_ is the critical value of *z* distribution, which is 1.96 at 95% confidence level; *σ* is the sample standard deviation; *n* is the sample size. The upper CI of original data is higher than that of smoothing data, indicating improved performance of the model trained by original data.





Figure S1. Sensor installation layout.


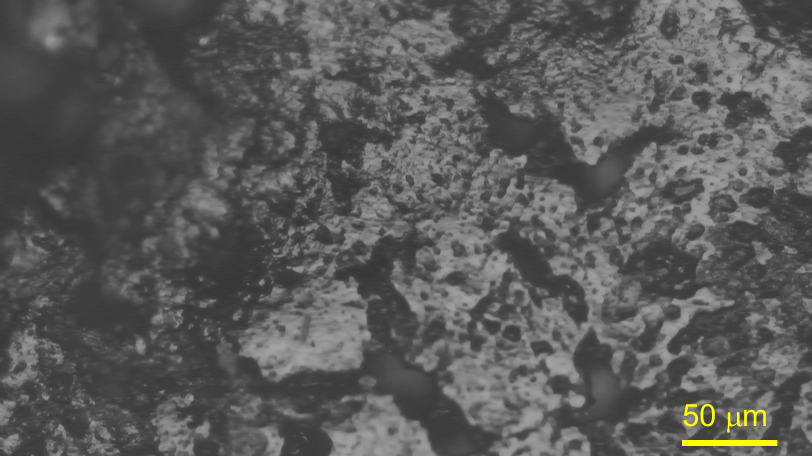


Figure S2. Surface profile of the positive tribolayer.


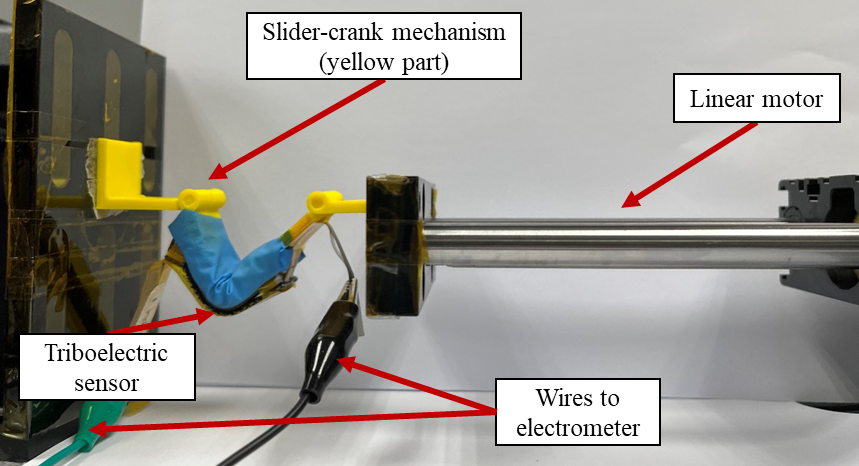


Figure S3. Experimental settings.


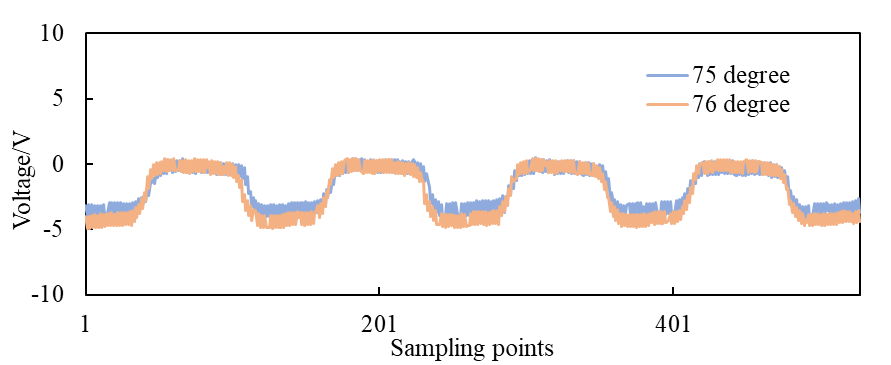


Figure S4. Voltage response change at bending angle of 75° and 76°.


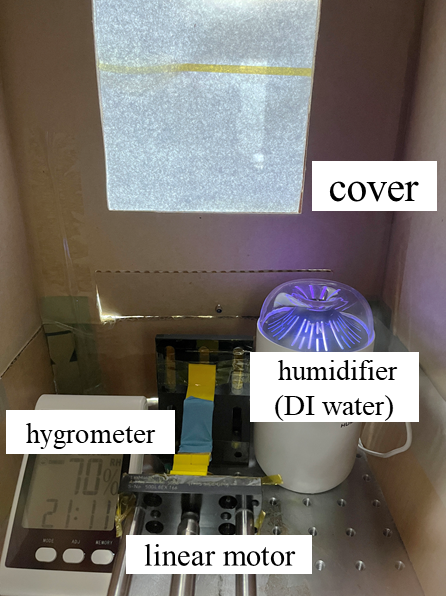

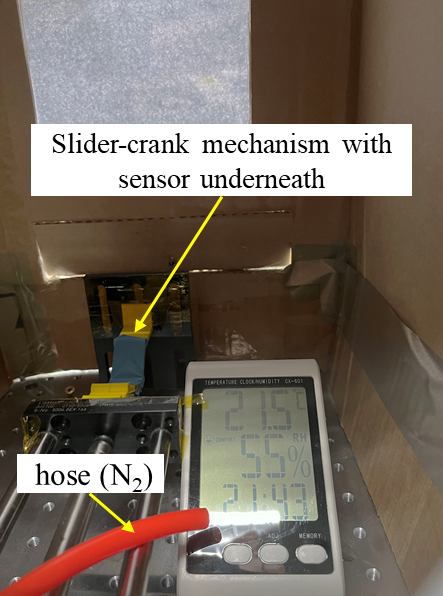


Figure S5. Experiment setup of relative humidity effects.


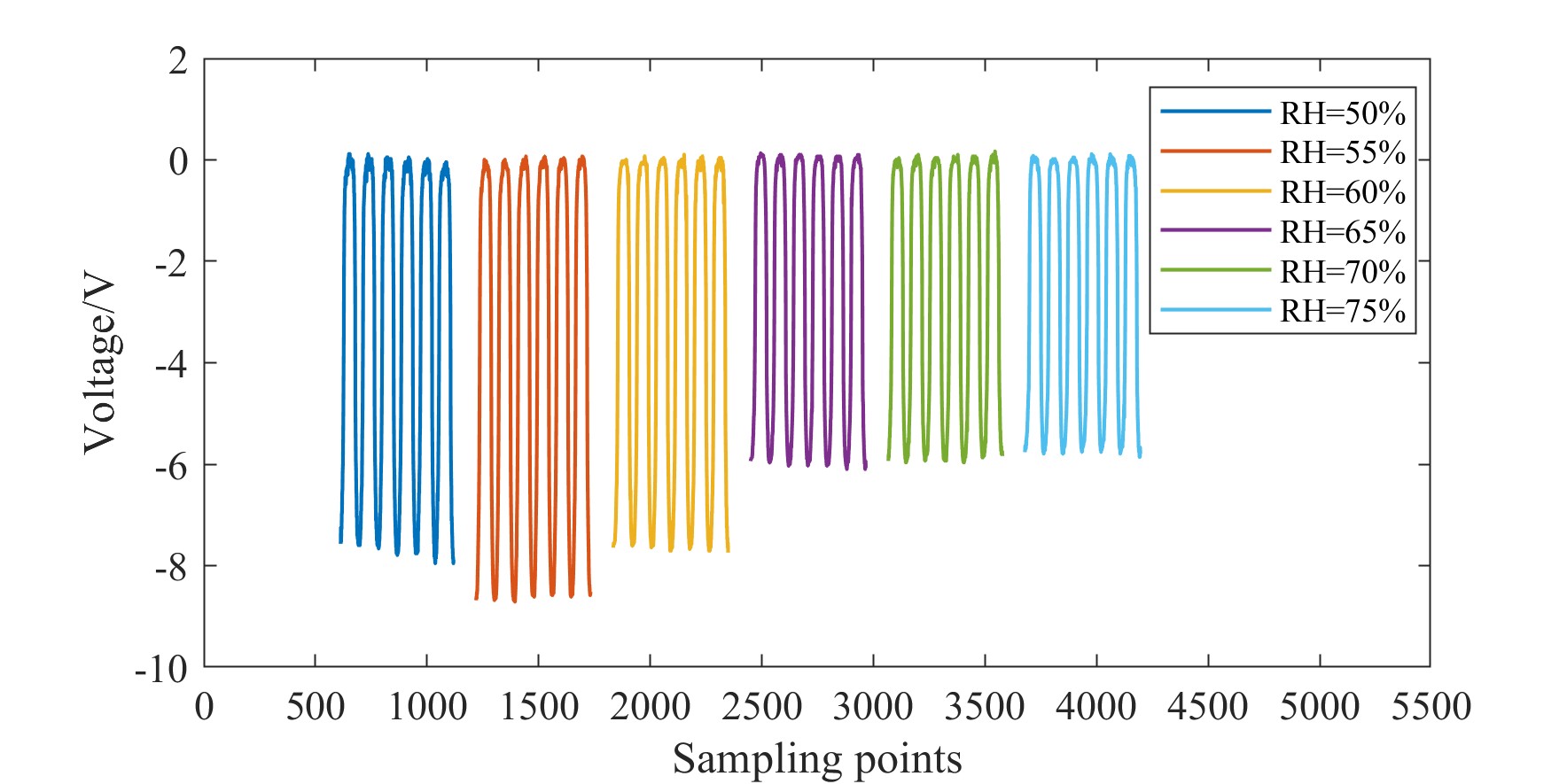


Figure S6. Voltage response at different RH (bending angle = 80°).


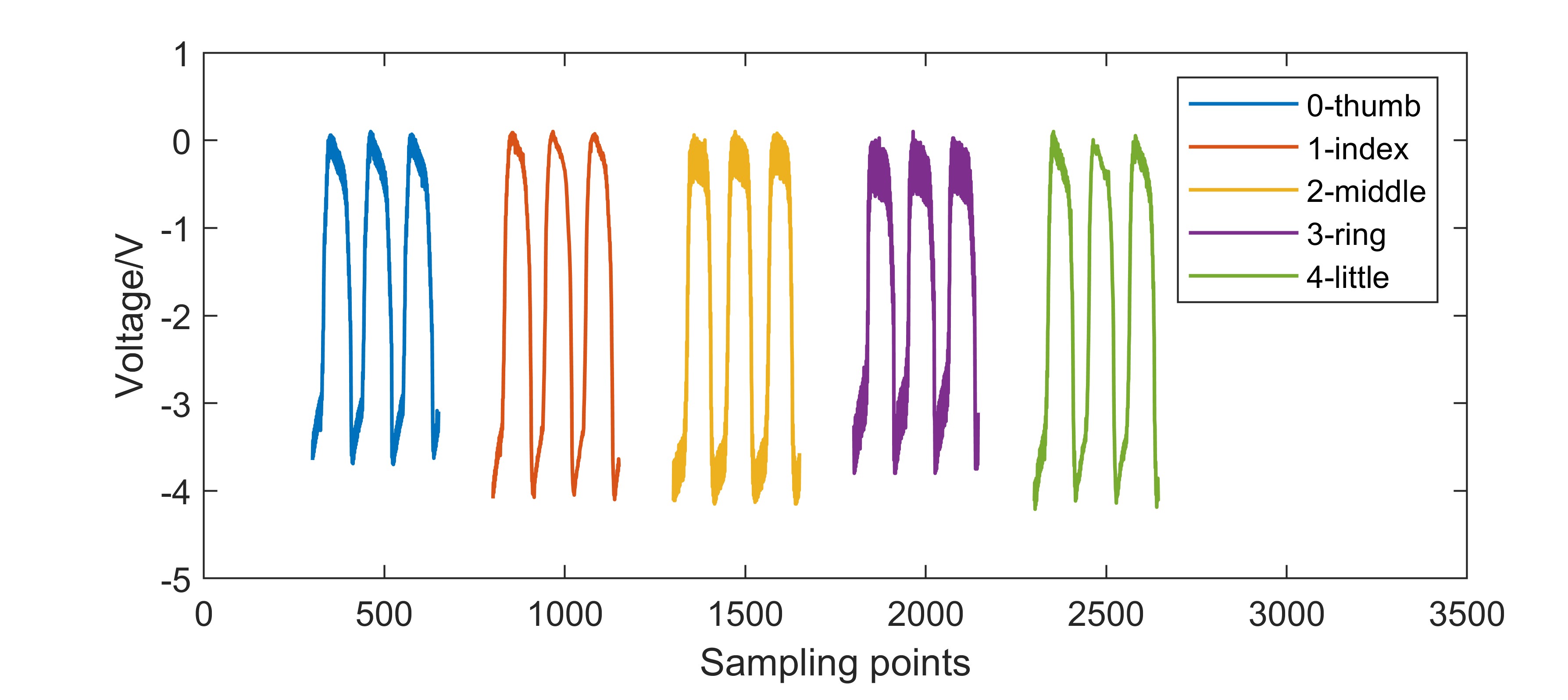


Figure S7. The five sensors’ voltage outputs (bending angle = 75°).


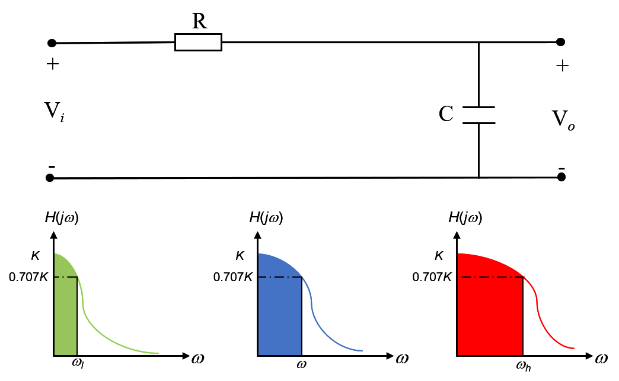

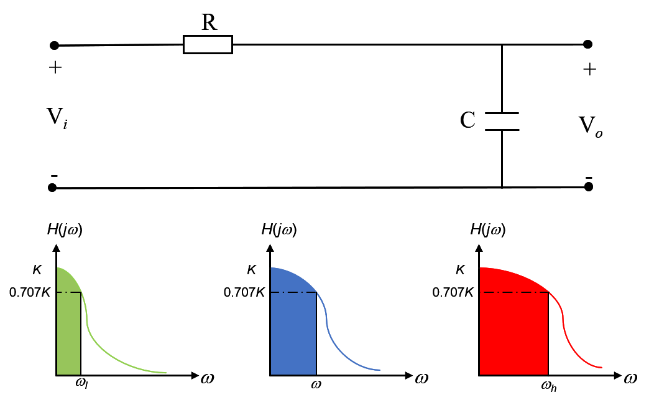


Figure S8. Schematic diagram of RC filter and its output characteristic in frequency domain at three different cut-off frequencies.


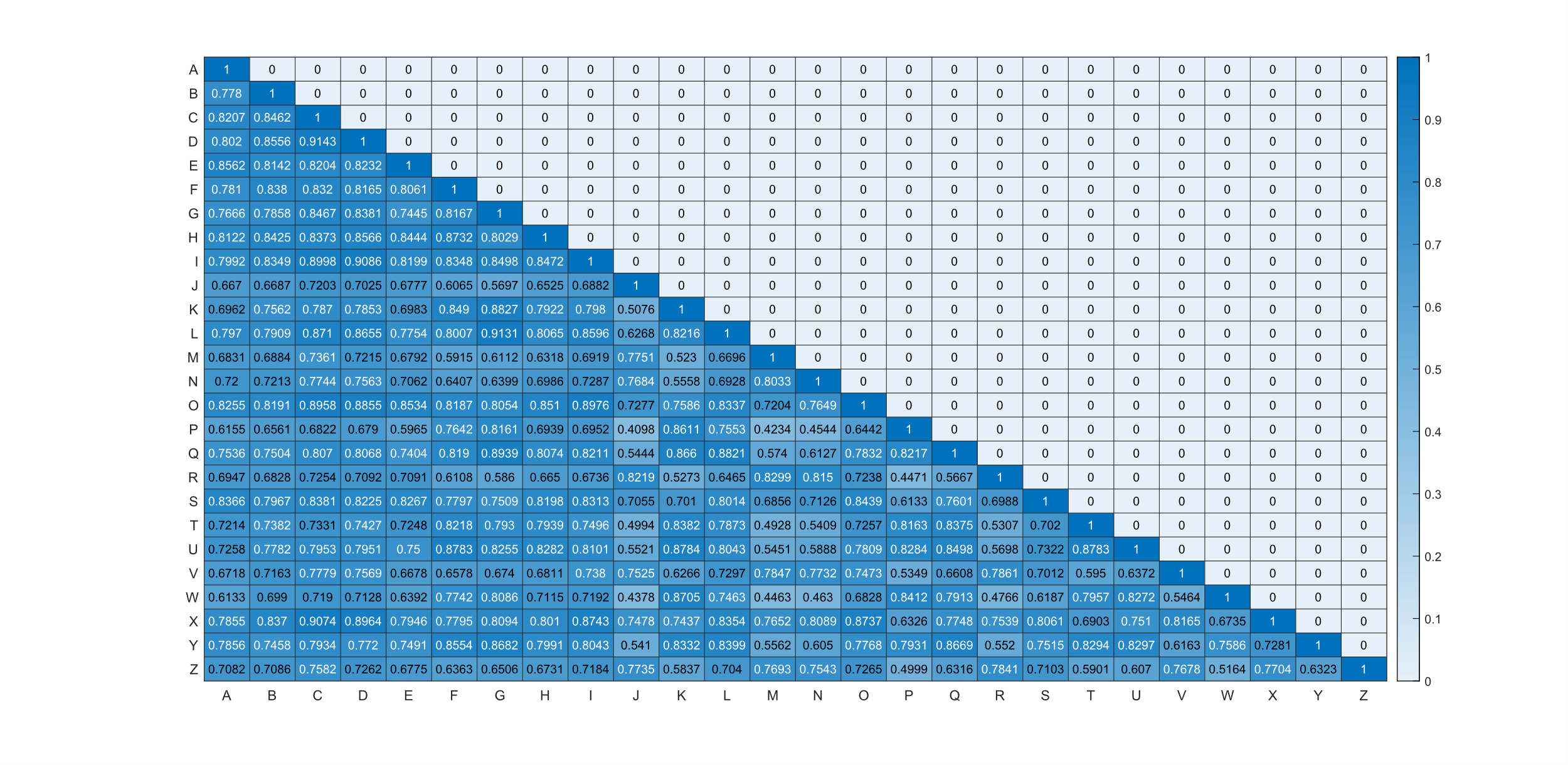


Figure S9. Correlation coefficient (CC) matrix.


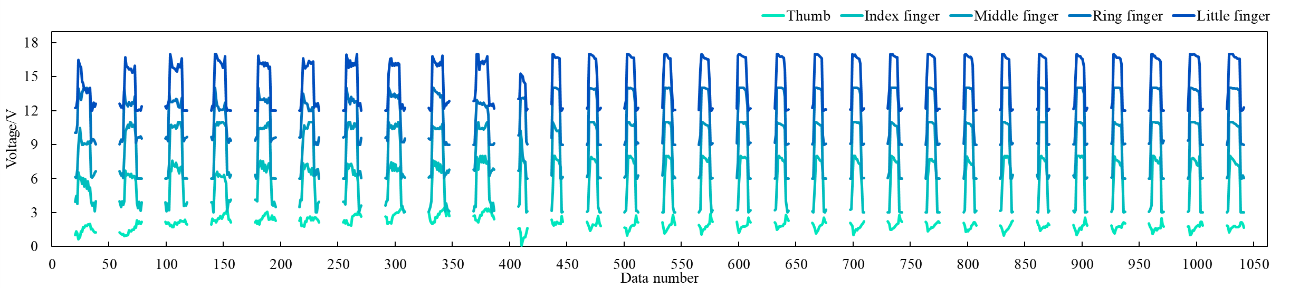

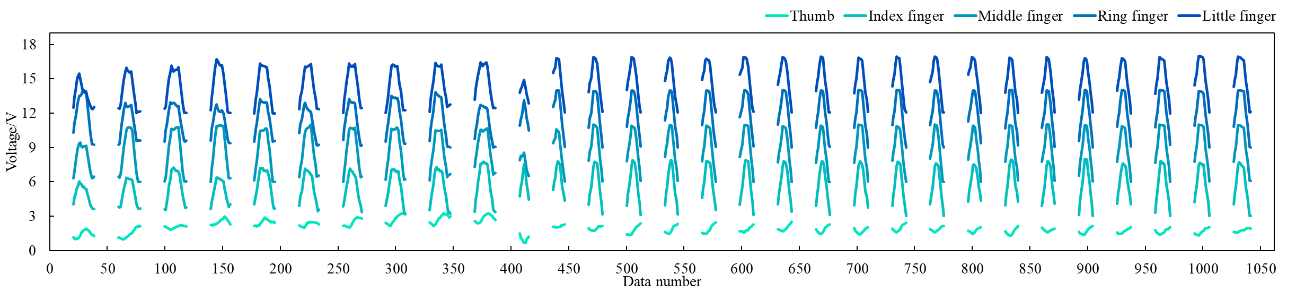


(a)

(b)

Figure S10. Signal patterns of letter A (a) before and (b) after data smoothing.


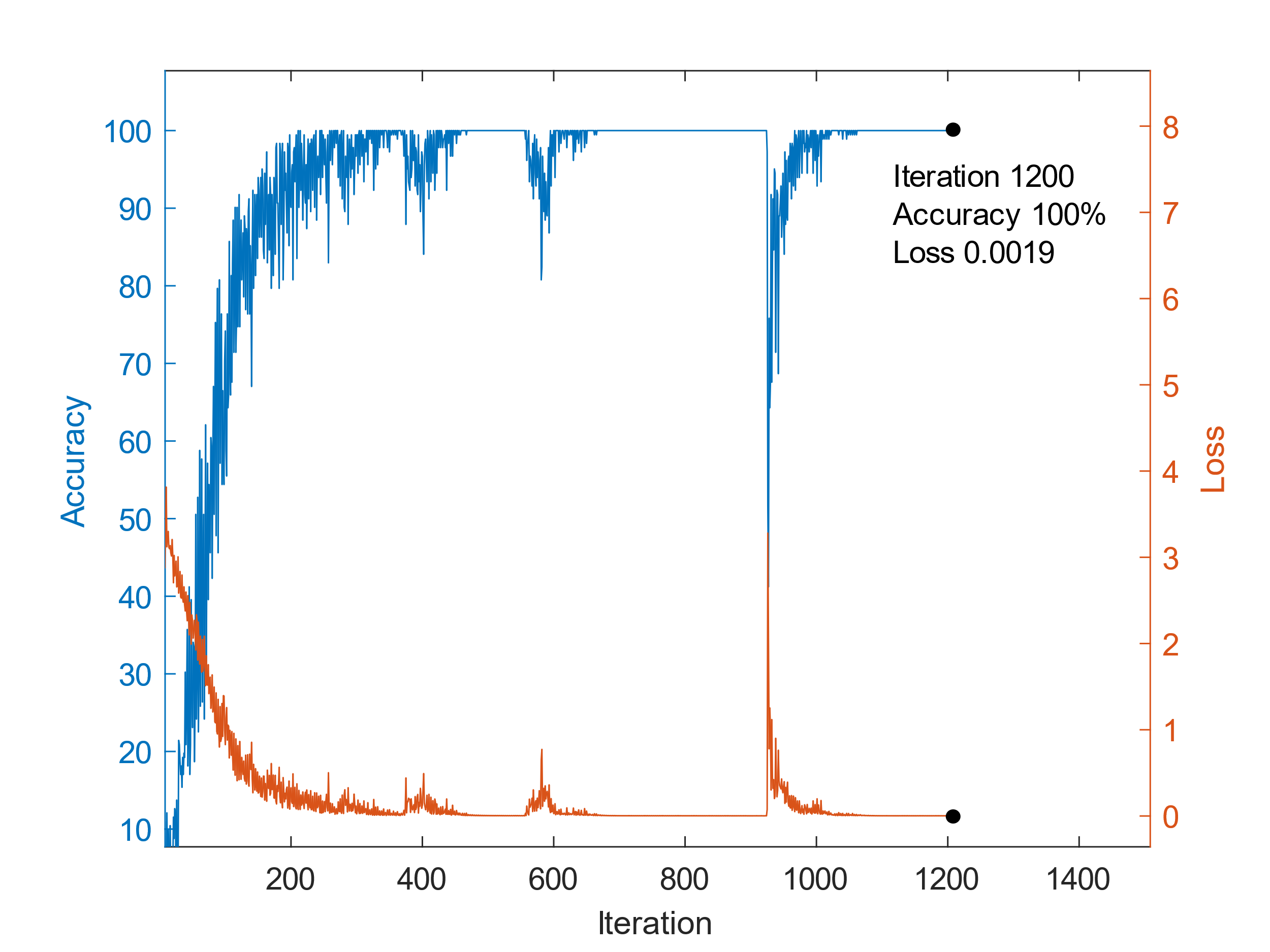


Figure S11. Training accuracy variations (original data).


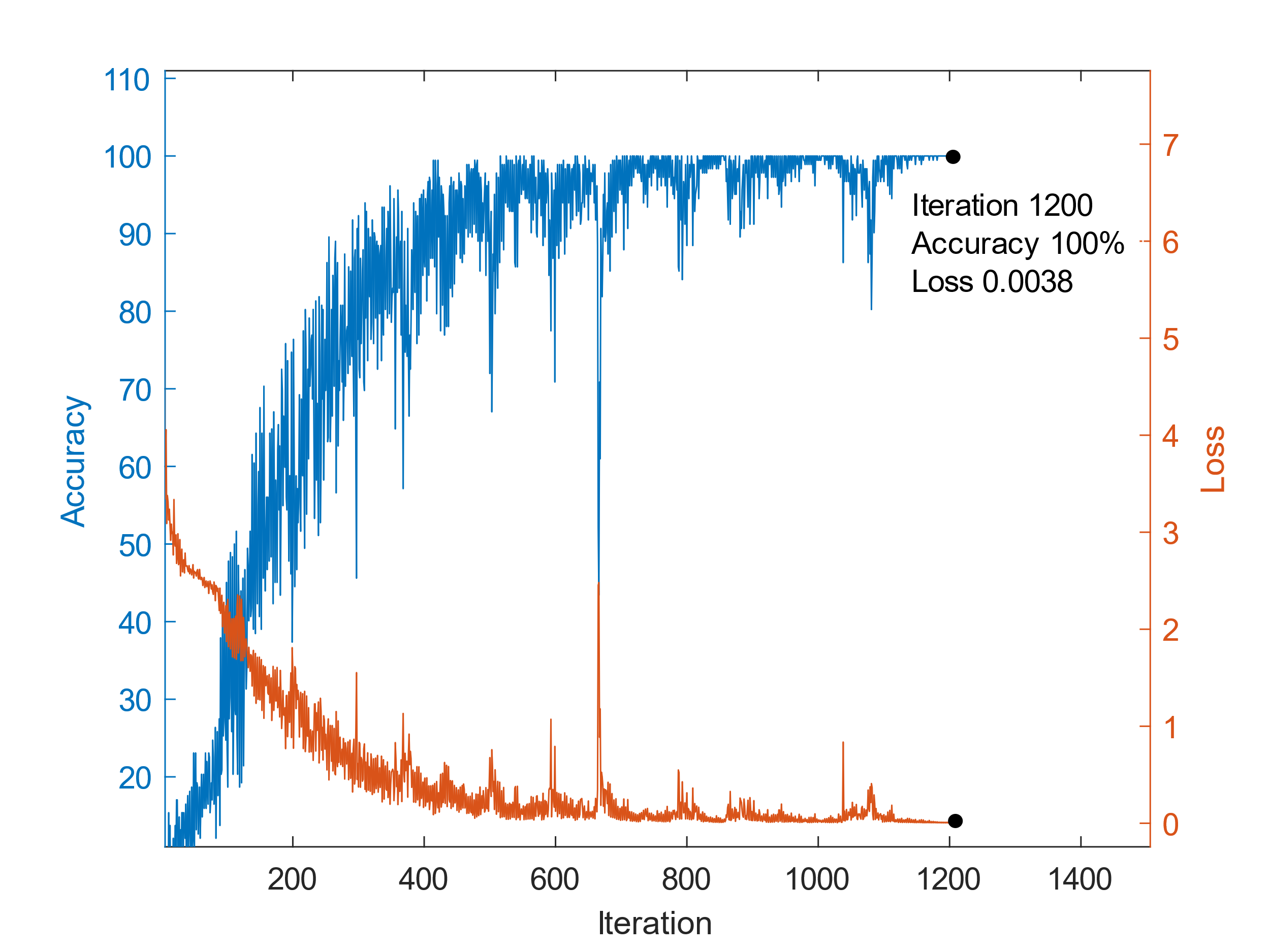


Figure S12. Training accuracy variations (smoothing data).

Figure S13. Comparison of the two models trained by two data groups.

**References:**

[1] X. Dan, X. Cao, Y. Wang, J. Yang, Z. L. Wang, Q. Sun, *ACS Applied Nano Materials* **2023**, *6* (5), 3590.

[2] H. Ouyang, J. Tian, G. Sun, Y. Zou, Z. Liu, H. Li, L. Zhao, B. Shi, Y. Fan, Y. Fan, *Advanced Materials* **2017**, *29* (40), 1703456.

[3] F. Cai, C. Yi, S. Liu, Y. Wang, L. Liu, X. Liu, X. Xu, L. Wang, *Biosensors and Bioelectronics* **2016**, *77*, 907.

[4] F. Wen, Z. Zhang, T. He, C. Lee, *Nature communications* **2021**, *12* (1), 5378.

[5] Y. Cao, H. Shao, H. Wang, X. Yang, Q. Gao, Q. Chen, J. Fang, T. Cheng, T. Lin, *Advanced Materials Technologies* **2022**, *7* (1), 2100830.

[6] Y. Luo, Z. Wang, J. Wang, X. Xiao, Q. Li, W. Ding, H. Fu, *Nano Energy* **2021**, *89*, 106330.

[7] R. Cao, J. Wang, S. Zhao, W. Yang, Z. Yuan, Y. Yin, X. Du, N.-W. Li, X. Zhang, X. Li, *Nano Research* **2018**, *11*, 3771.

[8] D. V. Anaya, T. He, C. Lee, M. R. Yuce, *Nano Energy* **2020**, *72*, 104675.

[9] C. Wu, T. W. Kim, J. H. Park, B. Koo, S. Sung, J. Shao, C. Zhang, Z. L. Wang, *ACS nano* **2019**, *14* (2), 1390.

[10] F. Wen, Z. Sun, T. He, Q. Shi, M. Zhu, Z. Zhang, L. Li, T. Zhang, C. Lee, *Advanced science* **2020**, *7* (14), 2000261.

[11] Z. Lin, Q. He, Y. Xiao, T. Zhu, J. Yang, C. Sun, Z. Zhou, H. Zhang, Z. Shen, J. Yang, *Advanced Materials Technologies* **2018**, *3* (11), 1800144.

[12] K. Dong, Z. Wu, J. Deng, A. C. Wang, H. Zou, C. Chen, D. Hu, B. Gu, B. Sun, Z. L. Wang, *Advanced Materials* **2018**, *30* (43), 1804944.

[13] Z. Zhao, C. Yan, Z. Liu, X. Fu, L.-M. Peng, Y. Hu, Z. Zheng, *Advanced Materials (Deerfield Beach, Fla.)* **2016**, *28* (46), 10267.

[14] P. K. Yang, L. Lin, F. Yi, X. Li, K. C. Pradel, Y. Zi, C. I. Wu, J. H. He, Y. Zhang, Z. L. Wang, *Advanced Materials* **2015**, *27* (25), 3817.

[15] W. Gong, C. Hou, Y. Guo, J. Zhou, J. Mu, Y. Li, Q. Zhang, H. Wang, *Nano Energy* **2017**, *39*, 673.

[16] H. Zhang, *Flexible and Stretchable Triboelectric Nanogenerator Devices: Toward Self‐powered Systems* **2019**, 19.

[17] W. G. Kim, D. W. Kim, I. W. Tcho, J. K. Kim, M. S. Kim, Y. K. Choi, *ACS nano* **2021**, *15* (1), 258.

[18] P. B. Shull, S. Jiang, Y. Zhu, X. Zhu, *IEEE Transactions on Neural Systems and Rehabilitation Engineering* **2019**, *27* (4), 724.

[19] G. Yuan, X. Liu, Q. Yan, S. Qiao, Z. Wang, L. Yuan, *IEEE Sensors Journal* **2020**, *21* (1), 539.

[20] B. G. Lee, S. M. Lee, *IEEE Sensors Journal* **2017**, *18* (3), 1224.

[21] Y. Lu, H. Tian, J. Cheng, F. Zhu, B. Liu, S. Wei, L. Ji, Z. L. Wang, *Nature communications* **2022**, *13* (1), 1401.

[22] Z. Zhou, K. Chen, X. Li, S. Zhang, Y. Wu, Y. Zhou, K. Meng, C. Sun, Q. He, W. Fan, *Nature Electronics* **2020**, *3* (9), 571.

[23] R. Wu, S. Seo, L. Ma, J. Bae, T. Kim, *Nano-Micro Letters* **2022**, *14* (1), 139.

[24] M. A. A. Faisal, F. F. Abir, M. U. Ahmed, M. A. R. Ahad, *Scientific Reports* **2022**, *12* (1), 21446.

[25] C. Savur, F. Sahin, in *2016 IEEE International Conference on Systems, Man, and Cybernetics (SMC)* IEEE, **2016**, 002872-002877.

[26] R. Fatmi, S. Rashad, R. Integlia, in *2019 IEEE 9th annual computing and communication workshop and conference (CCWC)* IEEE, **2019**, 0290-0297.

[27] P. Tan, X. Han, Y. Zou, X. Qu, J. Xue, T. Li, Y. Wang, R. Luo, X. Cui, Y. Xi, *Advanced Materials* **2022**, *34* (21), 2200793.

[28] Y. Liu, X. Jiang, X. Yu, H. Ye, C. Ma, W. Wang, Y. Hu, *Nano Energy* **2023**, *116*, 108767.

[29] X. Zhang, X. Chen, Y. Li, V. Lantz, K. Wang, J. Yang, *IEEE Transactions on Systems, Man, and Cybernetics-Part A: Systems and Humans* **2011**, *41* (6), 1064.

[30] R. Ramalingame, R. Barioul, X. Li, G. Sanseverino, D. Krumm, S. Odenwald, O. Kanoun, *IEEE Sensors Letters* **2021**, *5* (6), 1.

[31] C. Dai, C. Ye, J. Ren, S. Yang, L. Cao, H. Yu, S. Liu, Z. Shao, J. Li, W. Chen, *ACS Materials Letters* **2022**, *5* (1), 189.

[32] Z. Zhao, Y. Qiu, S. Ji, Y. Yang, C. Yang, J. Mo, J. Zhu, *Sensors and Actuators A: Physical* **2024**, *365*, 114877.
